# Does Relationship End Precede Cognitive Decline? An Analysis of the Health and Retirement Study

**DOI:** 10.1093/geroni/igab046.1092

**Published:** 2021-12-17

**Authors:** Douglas Hanes, Sean Clouston

**Affiliations:** 1 Stony Brook University, Stony Brook, New York, United States; 2 Renaissance School of Medicine, Stony Brook University, Renaissance School of Medicine, Stony Brook University, New York, United States

## Abstract

Relationship status is thought to be associated with cognitive health in older adults, with married persons performing better on memory assessments than unmarried-cohabitating, single, divorced, and widowed persons. However, questions remain about whether relationship termination causes cognitive decline, is a result of it, or whether they share a cause; and the mechanisms by which such a relationship might operate. To address this gap in the literature, we hypothesized that relationship termination could affect cognition via the following five pathways: (1) post-termination depression; (2) loss of distributed-cognition partner; (3) cognitive depletion from caring for partner in declining and ultimately terminal health; (4) divorce to preserve assets to qualify for Medicaid to cover healthcare for cognitive decline; and (5) post-termination changes in neuropsychiatric symptoms alongside a pre-existing neurodegenerative condition that also causes cognitive decline. Using data from the 2000–2016 waves of the Health and Retirement Study (HRS; N = 23,393), we found that relationship termination, whether due to divorce or widowhood, was associated with cognitive decline. Using mixed-effects regression we found that the rate of cognitive decline increased after relationship termination (widowhood: □ = -0.587, p <0.001; divorce: □ = -0.221, p <0.001), supporting mechanism (5). Using HRS data for respondents and their spouses’ mental and physical health, health insurance, and activities of daily living, we also find support for mechanisms (1) and (3). Relationship termination is a critical juncture in a person’s life course that has multiple implications and may, ultimately, worsen patients’ conditions.

